# Every child, every day, back to play: the PICUstars protocol - implementation of a nurse-led PICU liberation program

**DOI:** 10.1186/s12887-022-03232-2

**Published:** 2022-05-13

**Authors:** M. Waak, J. Harnischfeger, A. Ferguson, K. Gibbons, K. H. Nguyen, D. Long

**Affiliations:** 1 Child Health Research Centre, The University of Queensland, 62 Graham Street, South Brisbane, Queensland 4101 Australia; 2 Paediatric Intensive Care Unit, Queensland Children’s Hospital, South Brisbane, Australia; 3 Centre for Applied Health Economics, School of Medicine and Griffith Health Institute, Griffith University, Brisbane, QLD 4131 Australia; 4 Centre for Health Service Research, Faculty of Medicine, University of Queensland, QLD, Herston, 4006 Australia; 5 School of Nursing, Centre for Healthcare Transformation, Queensland University of Technology, Brisbane, QLD Australia

**Keywords:** ABCDEF bundle, Paediatric, Critical care, Post intensive care syndrome, Child

## Abstract

**Background:**

As admissions to paediatric intensive care units (PICU) rise and mortality rates decline, the focus is shifting from survival to quality of survivorship. There is paucity of internationally accepted guidelines to manage complications like over-sedation, delirium, and immobility in the paediatric setting. These have a strong adverse impact on PICU recovery including healthcare costs and long-term functional disability. The A2F bundle (ABCDEF), or ICU Liberation, was developed to operationalise the multiple evidence-based guidelines addressing ICU-related complications and has been shown to improve clinical outcomes and health-care related costs in adult studies. However, there is little data on the effect of ICU Liberation bundle implementation in PICU.

**Methods:**

PICU-STARS will be a single centre before-and-after after trial and implementation study. It is designed to evaluate if the multidimensional, nurse-led ICU Liberation model of care can be applied to the PICU and if it is successful in minimising PICU-related problems in a mixed quaternary PICU. In a prospective baseline measurement, the present practises of care in the PICU will be assessed in order to inform the adaptation and implementation of the PICU Liberation bundle. To assess feasibility, implementation outcomes, and intervention effectiveness, the implementation team will use the Consolidated Framework for Implementation Research (CIFR) and process assessment (mixed methods). The implementation process will be evaluated over time, with focus groups, interviews, questionnaires, and observations used to provide formative feedback. Over time, the barriers and enablers for successful implementation will be analysed, with recommendations based on “lessons learned.”

All outcomes will be reported using standard descriptive statistics and analytical techniques, with appropriate allowance for patient differentials in severity and relevant characteristics.

**Discussion:**

The results will inform the fine-tune of the Liberation bundle adaptation and implementation process. The expected primary output is a detailed adaptation and implementation guideline, including clinical resources (and investment) required, to adopt PICU-STARS in other children’s hospitals.

**Patient and public involvement statement:**

The authors thank the PICU education and Liberation Implementation team, and our patients and families for their inspiration and valuable comments on protocol drafts. Results will be made available to critical care survivors, their caregivers, relevant societies, and other researchers.

**Trial registration:**

ACTRN, ACTRN382863. Registered 19/10/2021 - Retrospectively registered.

**Study status:**

recruiting.

**Supplementary Information:**

The online version contains supplementary material available at 10.1186/s12887-022-03232-2.

## Key messages

Little is known on effective implementation of the Liberation bundle in critically ill children.

This hybrid mixed methods project will evaluate both the adaptation feasibility and effectiveness of the Liberation bundle.

The study will generate knowledge on how to improve PICU clinical practice and implement optimum care for children admitted to PICU.

### Strengths and weaknesses of this study

This will be the first comprehensive study to investigate the feasibility and impact of the A2F bundle adaptation to an Australian PICU.

The hybrid mixed methods study design will allow reporting of a detailed adaptation and implementation guideline, including clinical resources (and investment) required, to adopt the PICU Liberation bundle in other children’s hospitals.

The PICU Liberation intervention is not amendable to blinding of patients, family or clinicians or randomisation. The primary limitation is the single-centre design.

## Background

### Context

Focus in paediatric critical care (PICU) has shifted from mortality only to improving morbidity and long-term outcomes of survivors [1]. The risks of PICU related complications (ventilator-induced lung injury, immobilisation, delirium, oversedation) are well established [2–6]. They are associated with increased risk of ventilator associated pneumonia, intensive care acquired weakness, increased hospital length of stay, and mortality. Importantly, they can result in persistent functional disability, which affects the quality of life of PICU survivors [7, 8]. Physical, social, emotional, behavioural and cognitive impairments related to these issues may last for years after discharge and are now known as Post Intensive Care Syndrome (PICS) [5, 9]. With achieving a reduction in PICU-related mortality from 8 to 18% to 2.3–5% over the past 50 years, survivorship and PICS linked to the underlying disease and PICU-related complications have become key research and quality improvement areas [7–12].

### The A2F bundle and ICU liberation program

Adherence to integrated pain, agitation and delirium clinical practice guidelines using pharmacologic and non-pharmacologic approaches can prevent long term detrimental patient outcomes and long term (including economic) burdens on the family and society [13, 14]. Operationalising these guidelines, however, has remained a challenge for many ICU clinicians [15]. Different bundles have been tried in the adult context, but the only bundle approach proven to be effective and addressing all key areas is the A2F bundle. The A2F bundle is the central framework for an ICU Liberation program [16–19], which aims to improve patient outcomes by providing the right care – that is, starting the right treatments and stopping ineffective treatments.

The A2F bundle focuses on addressing over-sedation, prolonged mechanical ventilation, forced immobility, and family involvement, especially in mechanically ventilated patients [15]. It includes: **A**ssess, prevent, and manage pain, **B**oth spontaneous awakening and breathing trials, **C**hoice of analgesia and sedation, **D**elirium: assess, prevent and manage, **E**arly mobility and exercise, and **F**amily engagement and empowerment [20].

Two decades of cumulative research supports the A2F bundle implementation as a strategy to improve the quality and quantity of life of the critically ill. Large adult studies have demonstrated improved outcomes and shown a dose-dependent effect on exposure to benzodiazepines, shorter duration of delirium, less time on mechanical ventilation, fewer ICU and hospital days, and better functional dependence on hospital discharge and on follow up [15]. Bundling these evidence-based strategies helps to standardise care processes, reduce variation in practise, and increase communication among ICU teams. It guarantees that all bundle elements are applied to all patients in a consistent and suitable manner. Organizations have been encouraged to implement all bundle components to optimise therapeutic outcomes due to the synergistic impacts of the bundle components. To achieve full impact, it is recommended that healthcare providers consider using the bundle every day, in every patient admitted to the ICU. Of all the adult ICU Liberation programs that rely on the A2F framework, the nurse-led interdisciplinary approach has shown the most promising outcomes [18, 20]. Subsequently, the implementation of an ICU Liberation program for adults has moved toward the nurse-led interdisciplinary collaboration in some centers [20].

### Knowledge and implementation gap

To date, there is lack of evidence for adaptation of Liberation program implementation and clear dose-response relationship for the A2F bundle in PICUs. Feasibility has been suggested by early work in the north American PICU context [21, 22] as part of the ICU Liberation group of the Society of Critical Care Medicine (SCCM).

It is essential to generate further evidence of effectiveness of the A2F bundle and evidence of successful implementation of the Liberation program in PICU. A nurse-led, interdisciplinary approach to adaptation is promising and includes the family’s perspective [17]. This will ensure that the bundle elements target the right care and interventions for children and their families, and that these would be patient-important outcomes. PICU care is different to adult ICU care, in that children are especially vulnerable due to their developmental stages and abilities (both physical and cognitive) and have specific disease conditions not seen at other stages of life (e.g. congenital heart disease).

Therefore, we plan to adapt the ICU A2F bundle to the PICU. We will implement and evaluate the feasibility and effectiveness of the pilot PICU Liberation program in a large children’s hospital in Brisbane, Australia. The adapted A2F bundle will be a nurse-led, interdisciplinary PICU Liberation model of care, that can be implemented and contextualised in other children’s hospitals.

### Study aims

We hypothesise that adaptation of A2F bundle and implementation of the ICU Liberation program is feasible in the PICU context. Second, implementation of the pilot PICU liberation program will have an impact on incidence and effects of PICU related complications.

To test the first hypothesis, we will describe the process of adapting the A2F bundle to PICU. We will generate and describe the roll-out plan for the pilot PICU Liberation program, including the development and delivery of education and training resources, in addition to a discussion on unit specific and organisational requirements. To assess the feasibility, we will describe the PICU and hospital resources required for the successful adaptation and implementation.

Further, efficacy of A2F bundle adaptation to PICU will be described by reporting impacts of the PICU Liberation program on PICU related complications, family and staff satisfaction and creating a sustainable feedback process to determine further changes required over time.

## Methods

### Study protocol

This single centre prospective before-after hybrid trial-and-implementation quality improvement initiative will be conducted in a 36-bed mixed medical-surgical-cardiac PICU in a large children’s hospital in Brisbane (Australia) with approximately 2000 admissions per year. The adaptation, as well as the implementation process, will involve the children’s parents, carers, PICU clinicians and researchers.

Waiver of ethical approval and consent for this clinical innovation was obtained from the Children’s Health Queensland Ethics Committee as the intervention is a quality improvement project. This study will be performed in accordance with the ethical principles of the Declaration of Helsinki, ICH GCP for Guidance on Good Clinical Practice and NHMRC National Statement on Ethical Conduct in Research Involving Humans [23, 24].

Children will be identified by screening consecutive admissions to the PICU by the interdisciplinary research and quality improvement team, including PICU medical, nursing and allied health staff. Their data will be analysed if inclusion criteria are fulfilled and no exclusion criteria present. (Table 1).

**Table 1:** Inclusion and exclusion criteria

Inclusion criteria	Admission to study PICU
	≤18 years of age on admission
	PICU LOS ≥ 24 h
	Expected survival ≥1 year post PICU admission
Exclusion criteria	Paediatric Advanced Resuscitation Plan (PARP) actively enacted during admission
	​Severe chronic disability precluding PICU liberation program participation
	Minimally consciousness state on admission

#### Measurement of exposures

**Fig. 1:**
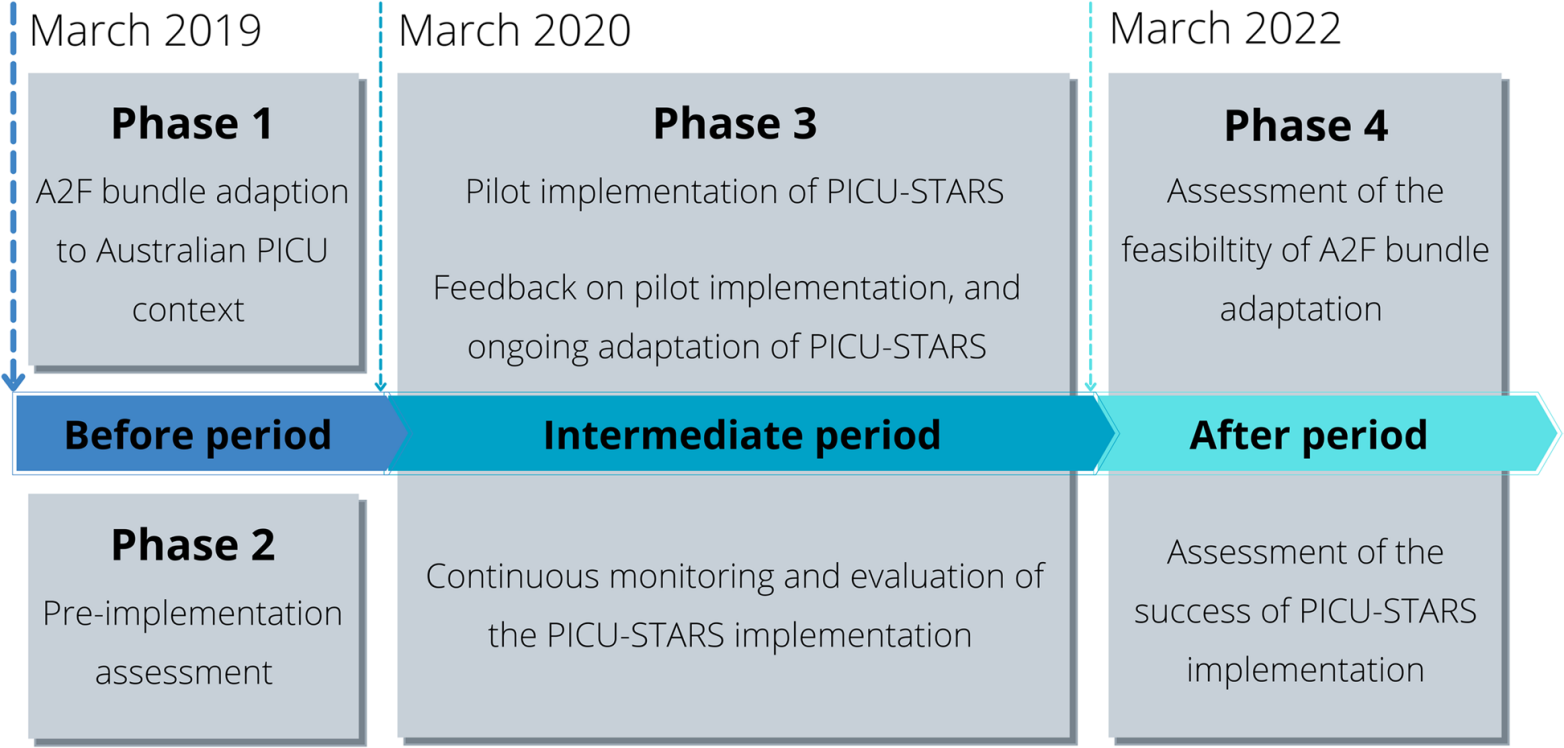
PICUStars study phases

The adaption phase began in March 2019 and is expected to be completed in 3 years. The period from March 2019 to March 2020 has been defined as the “Before period” including Phases 1 and 2; the period from March 2020 to March 2022 as the “Intermediate period” including Phase 3 (pilot implementation); the period from March 2022 as the “After period” signifying Phase 4 (feasibility assessment and impact), see Fig. 1 and Appendix 1.

The Consolidated Framework for Implementation Research (CFIR) will be used to guide the PICU Liberation bundle’s adaptation, implementation, and process evaluation. The CFIR is divided into five major domains: intervention features, inner and outer setting, individual characteristics, and implementation process. These domains have been founded to interact in rich and complex ways to influence implementation effectiveness [25]. The PICU Liberation bundle (the intervention) will be fully documented in a CFIR-based implementation “toolkit” before the end of the trial. The toolkit will outline the circumstances and resources needed for other children’s hospitals to adopt the bundle, as well as how to adjust bundle features to the environment, clinical team organisation, and workflows of different hospitals. During the intervention establishment phases, the study team and interested clinicians will collaborate to build and test an effective feedback loop and related clinical team response. (Phases 1–3).

### Implementation process

#### Phase 1: bundle adaptation to Australian PICU context

##### Objectives

To adapt the ICU Liberation A2F bundle to PICU through identification of specific requirements for children necessitating special consideration within the A2F bundle elements. To account for further neuroscientific developmental stages, each bundle element will then be adapted for infants ≤6 months of age in the “Baby-Liberation” program.

**Fig. 2:**
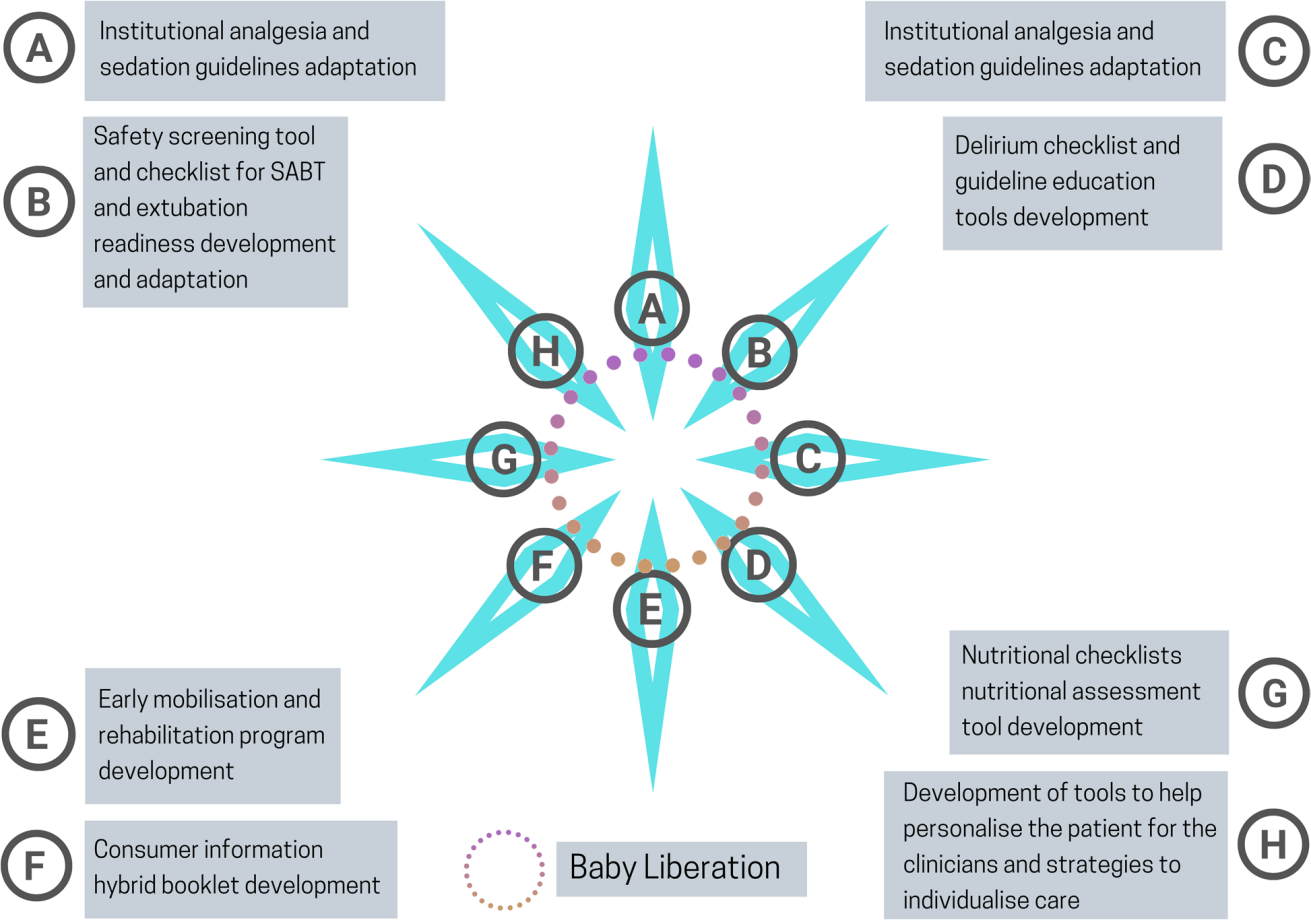
PICUstars bundle elements and educational tools

Activities:

- Comparison of current practice and Liberation model of care to identify gaps and areas for improvement required. Current assessment tools and documentation will be workshopped, and results will inform the development of the nurse-led, interdisciplinary PICU Liberation model of care.
- Comprehensive review of the current clinical assessment tools, guidelines and documentation (e.g., pain assessment, sedation assessment) to identify and remove duplicates, and to add updated and evidence-based assessment tools and guidelines where needed.
- A series of focus groups and workshops to discuss and gather feedback on the acceptability of the PICU Liberation bundle elements (see Fig. 2), and the proposed education tools developed by the PICU Liberation program implementation group.
- Assessment of any specific bundle requirement for use in specific patient cohorts; for example, neonates and infants less than 6 months of age.
- Regular assessment and revision (when appropriate) of the adapted PICU Liberation elements and contents will occur. A feedback and evaluation process will be established and include clinician and family feedback, audits, and evaluations (Table 2). Survey analyses and feedback from experienced clinicians that will be engaged to help improve each bundle element based on learnings from “provide care as required” will inform changes required as well.
- The four study phases will include parent/carer and clinician engagement workshops.
- Ongoing modifications to the PICU’s Clinical Information System to streamline documentation, to add assessment pieces where required for new content (e.g., early mobility assessment), and ensure all documentation is purposeful for clinical decision-making.

**Table 2:** Instruments and measures used to assess implementation feasibility and success (adapted from PUN et al.) [16]

**Instrument**	**Target population**	**Study phase**	**Outputs**
Empathic 30-AUS questionnaire	Parents of eligible children	From phase 2 onwards	Family engagement and satisfaction
PICU Liberation knowledge and perceptions questionnaire	PICU staff	Phase 2, 3 and 4	Drivers, barriers, knowledge gaps
Organisational readiness questionnaire	PICU staff, organisational leadership teams	Phase 2, 3 and 4	Drivers, barriers, leadership effectiveness, culture and employee morale or satisfaction/meaning making
Daily Goal checklist	PICU staff	Phase 3	Proportion of Liberation goals set
Check-in Audit	PICU staff	Phase 3	Drivers, barriers, knowledge gaps
Measure	**Definition**	**Study Phase**	**Outputs**
Feasibility	≥ 75% bundle compliance (daily goals set) at 1 year	Phase 3	Control charts of bundle compliance
Bundle fidelity (bundle compliance): Compliance with daily patient screening for PICU Liberation program using the daily goals checklist; eligible bundle element performed per patient day	≥ 75% absolute and/or ≥ 25% improvement from baseline compliance rate of checklist completion	Phase 3	Control charts of liberation goals set and bundle element activities performed per eligible patient day
Bundle performance	impact of bundle on process and quality of care (the practices that the bundle was designed to influence)	Phase 3	Complete performance is measured by a patient-day in which every eligible element of the bundle was performed (i.e., 100% of the bundle versus anything less) and “proportional performance” as the percentage of eligible elements a patient received on a given day (i.e., “bundle dose”). This will be measured only if the patient was in the PICU for a full 24 h (Table 4)

#### Phase 2: pre-implementation assessment

##### Objectives

To identify barriers and facilitators for the PICU Liberation program; to assess organisational readiness and parent satisfaction with current care practice; to collect baseline data of primary and secondary outcomes.

Activities:

- Assess barriers and facilitators for the PICU Liberation program using mixed methods, including targeted questionnaires, focus and expert group feedback.
- Assess knowledge and perception (acceptability) of the PICU Liberation bundle by staff as well as level of engagement of PICU clinicians.
- Assess organisational readiness (including teamwork and collaboration) and resource availability for implementing the PICU Liberation program (including education and training group activities, unit specific requirements including ability of the electronic medical record system to capture goals, and organisational readiness including culture and acceptability of concepts including inclusion of parents at ward rounds and other systems, settings and processes).
- Assess consumer engagement strategies and communication materials, ward round tools.
- Assess staff wellbeing (including retention).

To limit the Hawthorne effect, PICU staff will not be actively notified of the study during this time, nor will they be instructed on PICU Liberation [18]. Methods to measure baseline rates of correct assessments for pain, level of sedation, delirium, and occurrence of PICU related complications will be established (covering activity levels, over-sedation, withdrawal, delirium, PICU acquired weakness, ventilator free days, VAP, CLABSI, immobility, CAUTI, falls, mediation errors, PICU readmission, accidental line removal) (see Table 3).

**Table 3:** A2F bundle [16] vs PICU LIBERATION bundle element specifics and measures (adapted from PUN et al. where able) [16]

Bundle element	Adult A2F bundle	PICU LIBERATION bundle	Measures
A – Optimising analgesia	PAD guidelines - pain assessments using a valid and reliable instrument	Institutional analgesia and sedation guidelines • Stepwise introduction of analgesics first in agitated or distressed child • Assessment of pain at least fourth hourly, using the FLACC score [26, 27].	Instances of • FLACC assessments/opportunities • Appropriate stepwise use of pain medication (percentage) • WAT [28] assessments/opportunities • Withdrawal instances Opioid and benzodiazepine use Instances of expressed breast milk and sucrose utilisation for procedural pain relief (number of prescriptions and administration episodes) Empathic questionnaire results (carers' perception of child’s pain management)
B – spontaneous awakening and breathing trials	Guide for spontaneous awakening trial (SAT) if patients is receiving continuous or intermittent sedative infusions Guide for spontaneous breathing trial (SBT) if receiving mechanical ventilation	• Safety screening tool and checklist for SABT adapted for PICU to guide nurse-led SABTs • Safety screening tool and checklist for extubation readiness adapted for PICU to guide nurse enabled extubations	Instances of • SABT screening performed/opportunities, • SABT trials/opportunities, • Extubation assessments/opportunity, • Instances of nurse-enabled extubation/opportunity • Ventilator associated pneumonia • Failed extubations, accidental extubations requiring re-intubation within 1 h • Delay from extubation ready to time of extubation Hours/days without invasive ventilation Empathic questionnaire results (carers' perception of child’s spontaneous awakening and breathing trials)
C – Choice of sedatives	PAD guidelines - agitation/sedation assessments using a valid and reliable instrument.	Institutional analgesia and sedation guidelines – • Discourage use of sedatives, only second-tier treatment in the management of an agitated, ventilated patient; benzodiazepines, chloralhydrate and ketamine discouraged unless clinically indicated • Assessment of sedation, agitation or arousal recommended at least fourth hourly, using the Richmond Agitation and sedation scale (RASS) [29]	Instances of • RASS assessments/ opportunities • Sedation goal set/ opportunities • Sedation titration performed appropriately/ opportunities • Number of patients with “deep sedation” (RASS > − 2) • Instances of PTSS • Medication side effects recorded (opioids, benzodiazepines, ketamine, chloral hydrate) • Medication errors recorded • CLABSI and CAUTI • Accidental line removal Sedative use (by number of classes of sedatives used e.g., Benzodiazepines, chloralhydrate, ketamine) Empathic questionnaire results (carers' perception of child’s sedation management)
D – Early assessment and management of delirium	PAD guidelines - delirium assessments using a valid and reliable instrument	Institutional delirium checklist and guideline – • Guide to non-pharmacological (encouraged as first option) and pharmacological delirium management strategies. • Delirium assessment recommended at least daily, using the CAP-D^38^	Instances of • CAP-D assessments/opportunities, • Delirium identified (instances), • Appropriate management plan followed/opportunities (instances of non-pharmacological interventions targeting delirium, instances of pharmacological interventions targeting delirium) • sleep adjuncts utilised (e.g. day/night routine; swaddling/nesting) Empathic questionnaire results (carers' perception of child’s delirium prevention management)
E – Early mobility and rehabilitation	Guide to achieving mobility activities that were higher than active range of motion (i.e., dangling at edge of bed, standing at side of bed, walking to bedside chair, marching in place, walking in room or hall)	• A structured early mobilisation and rehabilitation program is commenced 24 h post admission to PICU. Children are classified into graded activity levels (Lizard, Koala, Wombat, Kangaroo) based upon the early mobilisation algorithm considering safety issues. • Assessment of physical function is completed by a paediatric physiotherapist, using the Children’s Chelsea Critical Care Physical Assessment tool (cCPAx) [30]	Instances of • Graded activity level goals set as daily goal, • Instances of mobility level sign on patient’s door • Mobility activities administered per patient per day • Mobility levels achieved: Lizard – immobile, routine positioning and range of motion unless contraindications; Koala – in bed activities including sitting; Wombat – in bedspace activities including mobility out of bed/standing; Kangoroo – mobility out of bedspace including ambulatory* • Falls Immobility Deconditioning (assessment of physical function by instances of Children’s Chelsea Critical Care Physical Assessment tool (cCPAx) Empathic questionnaire results (carers' perception of child’s mobility management)
F – Family engagement and empowerment	A family member/significant other was educated on the A2F bundle and/or participated in at least one of the following: rounds; conference; plan of care; or A2F bundle related care	Consumer information material educating family/carers on PICU Liberation bundle including goal setting and execution, family participation in cares, neurodevelopmental and early mobilisation activities and rounds	Instances of • Tools used to ensure family inclusion (e.g. Daily goals chart updated with goals set, likes/dislikes on “getting to know you” form utilised), • Family participation in liberation goal setting, • Family participation in liberation goals activity (education provided on PICU liberation, cares, neurodevelopmental and early mobility activity, rounds, plan of care including baby liberation flower, instances of therapeutic cuddles, instances of trips outside the patient’s room) • Family communication with healthcare providers “have you been kept up to date?” • empathic questionnaire completion/opportunity Number of Questionnaires administered to assess family coping and staff meaning making. Empathic questionnaire results (carers' perception of engagement/inclusion/respect/care)
G – Good Nutrition	Not part of adult A2F	Institutional nutritional checklists - Anthropometric assessments (actual weights rather than estimated weights), nutritional assessments recommended at least weekly, patient specific nutritional goals set. Paediatric growth charts completed. Oral feeding readiness assessments	Instances of • Patient weight assessment and weight estimates • Nutritional goals set, instances of nutritional goals achieved • Nutritional assessment tool used, • Weight obtained, instances of appropriate nutrition delivered (defined as 2/3 of requirements reached enterally or parentally from 48 h post I/V), deconditioning, ICU related weakness, cognition. • Nutrition free days. Referrals to speech pathology Oral feeding readiness assessments Nutrition delivery routes i.e. nasogastric, parenteral Empathic questionnaire results (carers' perception of child’s feeding and nutrition management)
H – Humanism	Not part of adult A2F	Institutional strategies to identify patients' and families' personal, developmental, and cultural preferences developed - family goals documentation recommended at least second daily.	Instances of • Humanism goals set (inclduing photos printed and displayed at bedside, “getting to know you” form/careplan) • Humanism goals achieved, instances of utilisation of tools to help personalise the patient for the clinicians (e.g. “getting to know you”), • Individualised care/opportunity, number and results of questionnaires administered to measure meaning-making for staff and family coping (control charts). • Family awareness of resources such as children’s book library, photo printing service (and use of same) • Completion of “getting to know you” form Empathic questionnaire results (carers' perception of individualised care; use of care planning etc)
Baby Liberation	Not part of adult A2F	Embedded in all PICU Liberation Bundle elements: Institutional strategies to ensure infant neurodevelopment is optimised – Baby Liberation goals documented on “flower” depicting categories of care such as family engagement, use of breast milk for mouth cares, nesting and swaddling etc.	Instances of Use of baby liberation flower use Use of adjuncts such as “zaky” hands, nesting, swaddling, cuddles Empathic questionnaire results (carers' perception of Baby Liberation programme)
Overall bundle performance	“Complete performance” defined as patient-day in which every eligible element of the bundle was performed (i.e., 100% of the bundle versus anything less) “Proportional performance” defined as percentage of eligible elements a patient received on a given day (i.e., “bundle dose” in %)	Additional performance measures: Target setting (Each morning Liberation targets including SP, RASS, mobility targets are to be set within the multidisciplinary ward round and documented in the PICU clinical information system) Targets reached/adjusted: each afternoon during “check-ins”	Measurement of instances of performance of each bundle element per patient day (only measured if the patient was in PICU for a full 24 h from d3 of PICU stay) Instances of daily goals set/opportunity. Instances of Liberation check-ins completed/opportunity Empathic questionnaire instances/opportunity Empathic questionnaire results (carers' perception of Liberation program and individual elements)

Mobility level examples: Lizard - routine positioning, range of motion activities; Koala - sitting in bed or on edge of bed, in bed cycling, other in bed mobility activities; Wombat - sitting out of bed, floor play, bed to chair transfers, short mobility activities, tilt table; Kangaroo - increased mobility activities around PICU and beyond, balcony visit, ride on toys

Semi-structured interviews and questionnaires will be utilised (Table 2). A purposeful sampling strategy will be used to select clinicians from each relevant profession including allied health, nursing, medical, education and administration. A variety of perspectives will be sought from key clinicians, organisation leadership and staff involved in the implementation of PICU Liberation. A similar process is repeated through Phases 3 and 4 of the study.

#### Phase 3 – pilot implementation with feedback and ongoing adaptation

##### Objectives

Implementation and adaptation based on adherence and effects including on PICU related complication rates.

While bundle adaptation will result in some variations, the PICU Liberation bundle is expected to have the following key components (see Fig. 2 and Table 3).

Activities:

- Implement PICU Liberation “check-ins” - a nurse-led, interdisciplinary rounding checklist - to ensure communication and application of all relevant bundle elements as clinically appropriate. The Plan-Do-Study-Act cycle will be utilised in the delivery of each bundle element, and adaptation will occur based on clinician and family feedback, and feasibility assessment following the initial roll-out to improve usability.
- Develop and implement a daily goals checklist for use during daily multidisciplinary ward rounds, which will include specific reference and reminders for each relevant bundle element.
- Provide education and training for PICU staff regarding the Liberation program via various mediums (new staff orientation, online, workshops, nursing education program curricula (theory modules and workbooks), individual bundle element in-services, bedside teaching, videos, breakfast sessions, case studies, simulations). This education will introduce the individual components of the bundle, use of assessment items, and recommendations for clinical actions based on assessment results. While education will be consolidated early in the implementation phase, it will be purposively staggered over all phases to ensure adequate updates and inclusion of new PICU staff. Education will be offered to nursing, medical, and allied health staff. Outreach and education to other hospital departments and services including non-clinical areas will be provided to ensure adequate understanding on downstream effects of PICU Liberation.
- Develop awareness of the relevance and importance of PICU Liberation program and its role in reducing PICU-acquired complications and improving short- and long-term outcomes.
- Repeat audits of A2F Bundle compliance, patient outcomes and satisfaction by parents as well as staff satisfaction and wellbeing assessments.
- Assess a) perceptions from staff about the PICU Liberation program and the implementation process; b) perceived barriers and facilitators to its implementation; c) how PICU Liberation has affected care; d) staff knowledge of key bundle elements and components, and e) perception of staff about the PICU Liberation educational resources. Note that the structural aspects (i.e., relative socioeconomic resources, political norms, policies) and their influence will not form part of this assessment.
- Continue to dynamically assess barriers and enablers to inform the continuous adaption of the A2F bundle operationalisation to improve the feasibility and implementation success.
- Focus group interviews with key stakeholder groups (i.e., family members, clinicians and administrative leaders) will be conducted to determine a) perceptions of PICU Liberation and the implementation process; b) perceived barriers and enablers to its implementation; and c) how PICU Liberation has affected care. All interview approaches will be used to maximise involvement, including face-to-face interviews, teleconferenced focused groups, and semi-structured interviews using separate interview guides. Families may be interviewed following exposure to the intervention and patient care experience (i.e., at or after PICU discharge). Clinicians may be interviewed mid-implementation to understand their impressions of the knowledge application process and adapt the implementation procedures if necessary.

Throughout Phase 3, the live reporting of selected compliance measures and patient outcomes will be visible to clinicians to provide motivation and real-time evaluation of current uptake of the A2F Bundle and their impact on the process and patient outcomes. Staff feedback will be proffered to consolidate compliance with PICU Liberation practices via on-the-spot feedback; compliment emails; patient and family narratives, photos, and videos included in weekly all-staff communications. Safety metrics will also be monitored and communicated to staff during this period.

#### Phase 4 - assessment of the feasibility of A2F bundle adaptation and the implement success of the PICU liberation program

##### Objectives

Assess feasibility, adherence, acceptability, and impact of pilot study roll-out including on PICU related complications.

**Fig. 3:**
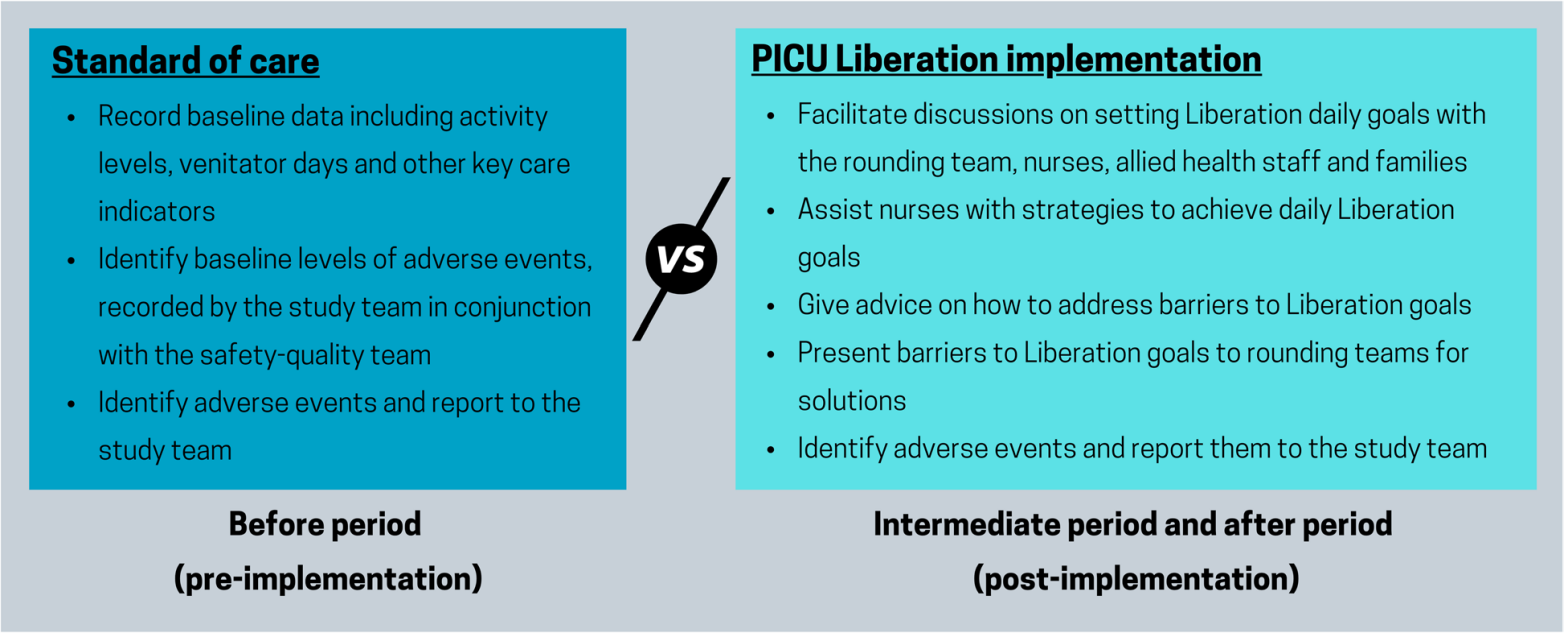
Interventions pre- vs post-Implementation of PICU Liberation (PICUstars)

Activities:

- Assess the feasibility of adapting the A2F bundles in PICU.
- Measure bundle fidelity (i.e. bundle compliance/adherence), using both quantitative analytic and qualitative methods (Table 2, Table 4). If a low degree of fidelity of certain PICU Liberation bundle elements is present, or concerns regarding fidelity are identified, modifications to the bundle elements will be considered to maintain the bundle integrity and primary objective of the PICU Liberation program, i.e., improve patient outcomes.
- Compare PICU-related complication rate between from pre- (before period) and post-implementation (intermediate and after periods, refer to Fig. 3).
- Conduct focus groups to ascertain learnings from the implementation process and gather feedback from PICU staff about successes and areas for improvement to inform further adaptation, if required.

**Table 4:** Definitions of PICU Liberation program: Bundle Performance and Daily Goals (adapted from PUN et al.) [16]

Element	Days eligible	Performance in the last 24 h it was documented that the patient received	Performance in the last 24 h it was documented on the daily goal checklist
A	All days	≥ 6 pain assessments using the FLACC assessments [26, 27]	FLACC goal
B1	Only days when patient received continuous or intermittent sedation	A spontaneous awakening trial (SAT) if receiving continuous or intermittent sedative infusions OR sedation target set as “light” in the daily goals	Suitability for SAT if receiving continuous or intermittent sedative infusions OR suitability for sedation goal set as “light”
B2	Only days when patient was on ventilatory support	A spontaneous breathing trial (SBT) if receiving invasive ventilation	Suitability for SBT if receiving invasive ventilation
B3	Only days when patient was on ventilatory support	An extubation assessments if receiving invasive ventilation	Suitability for extubation assessments if receiving invasive ventilation
C	All days	≥ 6 agitation-sedation assessments using the RASS (Richmond Agitation-Sedation Scale [29]	RASS goal
D	All days	≥ 1 delirium assessments using the CAP-D [31]	CAP-D requested
E	Only days when patient was not classed as “lizard – for range of motion activities only”	≥ 1 Mobility activity administered that was higher than range of motion	Mobility goal (Lizard – passive range of motion unless contraindicated; Koala – in bed activities including sitting; Wombat – out of bed activities including transfers; Kangaroo – activities away from bedspace including ambulatory) set
F	Only days when family was present	Family member/carer educated on the PICU Liberation bundle and/or participated in at least one of the following: liberation goal setting, cares, neurodevelopmental or early mobility activity, rounds, plan of care.	Suitability of family inclusion in liberation goal setting, cares, neurodevelopmental or early mobility activity, rounds, plan of care.
G	All days	Nutritional assessment tool used OR weight obtained	Nutritional goal set
H	All days	Tool to help personalise the patient for the clinicians administered OR humanism activity administered	Humanism goal set
Baby Liberation	Only infants	“flower” tool to adapt each bundle element to include developmentally appropriate components	Baby Liberation goals documented

During Phase 4, each bundle element will be continuously adapted based on feedback and feasibility assessment following the initial roll-out to improve usability. As a proof of concept for wider adoption in other centres, we will embed evaluation metrics within each of the educational formats provided. All the measures that reflect patient outcomes, patient and parent experience, and compliance will be collected, monitored, and communicated to staff well beyond the implementation period to ensure ongoing adherence to the PICU Liberation bundle.

### Statistical analysis

#### Sample size

The study follows a prospective before-after hybrid trial-and-implementation approach, whereby efficacy data will be collected in a convenience sample of 1800 patients (estimated) and implementation and process data will be collected from PICU staff and parents over the three-year period.

This relatively large sample of 1800 instances of complete or partial bundle implementation effects will allow an in-depth analysis of the methods considering the relatively large heterogeneity of the study population.

#### Data collection

Data will be collected for the periods of before (pre-implementation), intermediate (during implementation, 12 months in), and after (post-implementation) as well as continuously through all study phases. Data collection will include bundle feasibility, impact and implementation measures as well as clinical data. (Tables 1, 2, 3, 4).

#### Descriptive statistic analyses

The twelve pre-implementation-months will be collated and presented as a single timeframe; all months during the implementation period will be described separately. Categorical variables will be described as counts (percentage). Continuous variables will be described by their median (interquartile range). Continuous outcome measures such as the length of stay (LOS) and hospital free days will be analysed using either survival analysis and competing-risks regression. The availability of recorded data on a specific characteristic will be described (e.g., we can only describe sedation on days which had any sedation data recorded).

In addition to analysis of the whole patient cohort, we will undertake sub-analyses for patients < 6 months of age, PICU stay shorter than 48 h and non-ventilated patients.

#### Impact measures of the PICU liberation program implementation

Orchestrated Testing will be used to assess implementation success, the impact of the PICU Liberation intervention, and identify essential components for best practise [32]. A clear implementation plan and structure for interaction are two of the Orchestrated Testing requirements. (generated in study phase 1, see Fig. 1 and Appendix 1). In our study, the number of bundle components in Phase 1 represents existing practise and Phase 4 represents bundle implementation, hence a Factorial (or Fractionated factorial) matrix is critical. In step 4, the results will be analysed. The ability to duplicate the study findings will be tested internally (repeat plan-do-act cycles) and externally, as part of a proposed secondary study testing bundle implementation at a separate PICU in Australia.

**Fig. 4:**
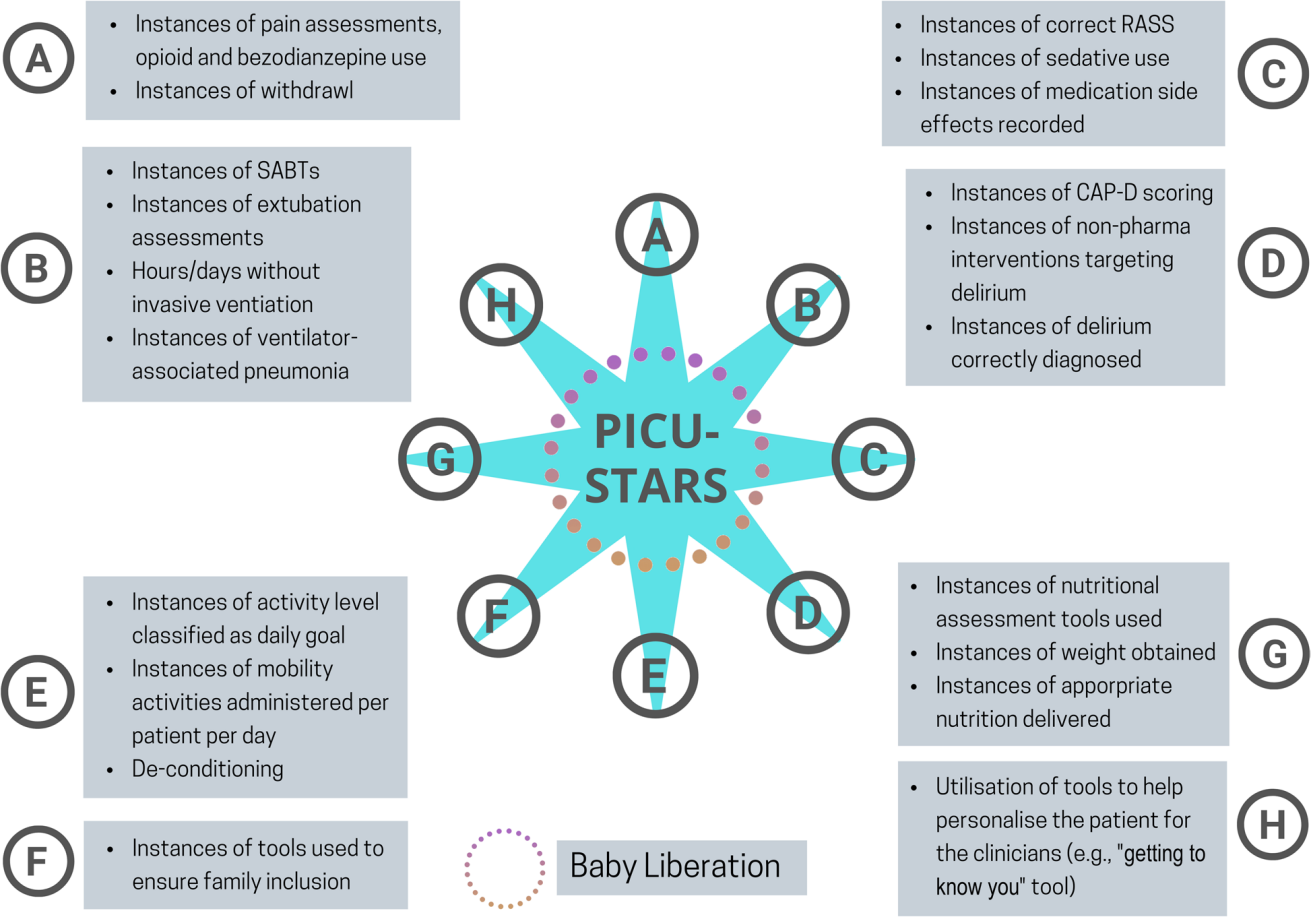
PICUstars bundle element outcome measures

Control charts depicting the PICU Liberation metrics will be used to track exposure to the intervention/consistency. (as described in Table 3 and Fig. 4 below).

Control Charts [33] will be used in repeated measure studies for bundle compliance and performance over time since they are an efficient way of measuring practise change in real-time. In reaction to positive or negative change, education and implementation tactics can be tailored. The control chart rules will be used to specify improvement [33, 34].

Before and after implementation, the process of care outcomes will be assessed. Background variables that may act as confounders in the process outcomes will be monitored (e.g., acuity, staffing ratios). The effects of each element and their combinations will provide preliminary estimations of the PICU Liberation bundle’s clinical significance. Absolute and relative risks, followed by logistic regression algorithms to adjust for relevant prognostic risk factors for dichotomous outcomes, will be calculated to assess secondary efficacy outcomes before and after deployment of the bundle. The incremental cost of PICU-related problems, as well as incremental cost effectiveness ratios and 95% confidence intervals, will be estimated for the economic analysis plan [35].

### Ethics and dissemination

#### Data management and oversight

The study investigators will be in charge of overseeing the project’s day-to-day operations and ensuring that the ICH-GCP criteria are followed. The data will be monitored by members of the PICUstars research team. Protocol adherence, effective study management, and timely completion of research procedures will all be monitored. On-going surveillance and adherence to the study protocol (intervention fidelity) will be monitored by the principal Investigator (PI) and clinical research nurse (CRN) during monthly audits.

Streamlined data collection instruments and procedures will be used. All other data will be collected by the CRN onto the case report form (CRF) directly from the source data. Data will be entered into a custom-build electronic database developed using the electronic data platform REDCap, hosted by Griffith University [36, 37].

#### Data storage and security

Identifiable data will be stored on institutional network drives, which will be protected by firewalls and other security measures. Hard copies of records will be kept in a locked cabinet in a safe place.

#### Only study personnel will have access to records and data. The data from the study will be de-identified, and a master linking log containing identifiers will be preserved and stored separately from the data. Dissemination

Results will be made available to the funders, critical care survivors and their caregivers, the relevant societies, and other researchers.

## Discussion

The protocol for a single-centre prospective implementation of evaluating the PICU Liberation programme, which adapts the A2F bundle in adult ICU to the PICU of a major children’s hospital in Queensland, is presented in this publication. The goal of the study is to determine the feasibility of adaptation, as well as to evaluate the PICU Liberation trial’s implementation success by assessing the capacity to meet A2F bundle objectives, improve patient quality of care, and optimise children’s recovery while reducing PICU length of stay. Up to 66% of critically ill children can develop PICU-related problems [11]. They not only have a short-term influence on hospital length of stay and cost of care, but they can also cause morbidity, which has long-term detrimental consequences for the child’s quality of life, such as psychiatric, behavioural, and neurocognitive issues (long-term impacts) [11]. There are no paediatric-specific bundles available to aid in the detection and prevention of PICU-related problems [38]. More studies that change from a mortality focus to a quality of survivorship focus are critical [3, 11, 39, 40].

The PICU Liberation project focuses on improving care quality through interdisciplinary team collaboration and best practises for long-term quality initiative implementation. In the adult ICU, there is substantial clinical evidence that the ICU Liberation programme improves patient outcomes significantly and dose-dependently [38].

For a successful implementation, there are necessary conditions: an evidence-based implementation framework, a context appropriate implementation framework, an implementation team leader or facilitator, inter-professional team engagement (nursing, allied health, medical, family), and the ability to customise PICU Liberation to the site needs. This project is designed with these conditions primed. First, it is guided by the Consolidated Framework for Implementation Research (CFIR), which has been shown to facilitate successful bundle adoption and improve the quality of care in adult and paediatric ICUs [25–27, 41]. It will also be guided by the quadruple aim: patient outcomes (including children and family satisfaction), staff satisfaction and work-related wellbeing, cost effectiveness in care, and population health) [28]. Second, several publications go into great detail about the Liberation package, and its implementation has been closely observed by one of our team members overseas, as our content expert and implementation team leader/advisory role. Third, certain aspects of the bundle are ‘adaptable,’ meaning they can be changed to fit a specific situation without jeopardising the intervention’s integrity. We have developed several working groups to adapt and review each of the Liberation bundle elements to ensure feasibility and optimal compliance. Lastly, we have foreseen the main causal factors that influence implementation outcomes (at structural-, organizational-, patient-, provider-, and innovation-levels).

This study is unique in that it includes all aspects of a multifaceted nurse-led PICU model of care in a large cohort of critically ill children, as well as measurement of important clinical outcomes aimed at shifting ICU culture away from the harmful inertia of sedation and restraints and towards an animated PICU filled with patients who are awake, cognitively engaged, and mobile, as well as family members engaged as partners with the PICU team at the bedside. Its advantages include its applicability, clinical team focus, and use of currently accessible resources. PICU Liberation study constitutes a clinical innovation. It was created by the interdisciplinary team and written concisely for intuitive adaptation. The study facilitators will ensure consistency and feasibility of goal setting and Liberation interventions as well as tracking children/s’ and families’ progress. It is designed to be a low tech, high yield clinical and rehabilitation intervention bundle that optimises care through a community of practice and team collaboration, that can be applied to every critically ill child, every day.

The findings of this study will add to our understanding of how to effectively implement optimal care for critically ill children. The research findings will be externally valid and should drive clinical application of the bundle in any PICU because this knowledge will be generalisable to the broader PICU population. Project outcomes and learnings will add to the general body of knowledge about implementation science in the PICU.

The Liberation algorithms allow nurses to readily contribute to goal setting on rounds and provides structure for the nurses to “liberate” their patient. While we believe that the Liberation bundle makes effective use of current resources, we recognise that transferring resources to Liberation interventions may have opportunity costs in certain scenarios. Owing to the nature of this trial-and-implementation health service research, there are notable limitations. The primary limitation of this study is the single-centre design. Given our institution’s standing as the state-wide referral centre, external validity will be achieved by designing the implementation pathway for adaptability in other PICUs locally and nationally.

Second, the PICU Liberation program is not amendable to randomisation or blinding of patients, family, or clinicians. The achieved Liberation assessments and interventions will be recorded by the bedside nurse who is unblinded and part of the intervention. The PICU Liberation facilitator will record achieved Liberation interventions during regular afternoon Liberation check-ins.

Third, to achieve the daily Liberation targets, we do not add additional people resources. This is a setting where current mobilisation, sedation, and ventilation goals are occasionally difficult to achieve. The Liberation bundle, we feel, is complementary to nursing philosophy and will be seamlessly integrated into their system with minimal, if not positive, impact on their workload. It is acknowledged, however, that this budget-neutral approach may not enable effective operationalisation of PICU Liberation, and a dedicated Liberation clinical team may be necessary.

Lastly, successful bundle adoption will create a culture shift from immobilised, sedated patients with limited family presence to comfortable awake patients who are cognitively engaged, and mobile with family members. This highlights a further limitation as bedside nurses become more comfortable applying those principles to all their patients over time, effect size may vary and increase over time, which may lead to a type II error. The high sample size and long post implementation measurement phase of 12 months may help to minimise this.

### Methodological limitations

Our prospective study design in which variates are reliably measured over time will provide stronger evidence for feasibility of this PICU Liberation implementation project than could be obtained from a retrospective design or offline assessment models.

The principal drawback of this study is its single-center design and the possibility of missing data (data failure), which would call into question the internal and external validity of the presented findings. Our research team, on the other hand, has substantial experience with excellent recruitment rates and data integrity in past trials of critically ill children receiving innovative therapies. Strategies to minimise missing data will include experienced study personnel with appropriate training and support to ensure accurate and timely capture and entry of data, streamlined IT solutions and utilisation of standardised database tools (REDcap).

## Conclusion

This provides a description of our study protocol and analysis plans for the PICU Liberation Trial. This project is aimed to maximise the efficiency of existing resources without requiring any new employees or funding, in addition to examining the benefits of goal-directed Liberation bundle adaptation on clinical outcomes and survivability. This study offers a way to utilise goal-directed Liberation interventions to enhance PICU clinical outcomes while reducing hospital costs.

## Supplementary Information

**Additional file 1.**

## Data Availability

The datasets used and/or analysed during the current study are available from the corresponding author on reasonable request.

## Abbreviations

PICU: Paediatric Intensive Care Unit
A2F bundle: ABCDEF bundle
CIFR: Consolidated Framework for Implementation Research
PICS: Post Intensive Care Syndrome
SCCM: Society of Critical Care Medicine
VAP: Ventilator Associated Pneumonia
CLABSI: Central Line-associated Bloodstream Infection
CAUTI: Catheter-associated Urinary Tract Infection
REDcap: Research Electronic Data Capture
