# Supplementary material for: Every child, every day, back to play: the PICUstars protocol - implementation of a nurse-led PICU liberation program
Source: BMC Pediatr. 2022 May 13;22:279. doi: 10.1186/s12887-022-03232-2 (PMC9102243; doi:10.1186/s12887-022-03232-2)
Supplement: Supplementary file 1 — Additional file 1. [file 12887_2022_3232_MOESM1_ESM.pdf]

SUPPLEMENTARY DOCUMENT (APPENDIX)

Implementation and timeline

Table A1. PICU Liberation (PICUstars) implementation plan and timeline

Steps	Implementation process	Timeline
**Engage (Phase 1)**	• Establish the inter-professional team (RN, MD, Pharmacy, allied health).<br>• Include patient and family representatives, and research team members. Clarify roles and processes.<br>• Define problems, set goals and timelines.<br>• bundle adaptation to local PICU Australian context.<br>• Comprehensive assessment of impediments to bundle and Focus groups feedback on feasibility of proposed bundle developed by implementation group.<br>• Assess local practice and resources.<br>• Systematically assess enablers and barriers to implementation of the bundle using evidence-informed strategies and measures from the KT literature.<br>• Develop implementation protocol.<br>• Tailor PICU Liberation Bundle elements to the needs of the PICU.<br>• Develop a process for reliable Bundle delivery to all patients.<br>• Match Liberation interventions and processes to mitigate challenges identified in assessment phase.<br>• Determine evaluation strategy.<br>A2F bundle adaption to the Australian PICU context.	6 months
**Educate (Phase 2)**	• Train operational leads on all intervention activities.<br>• Train front line clinicians and stakeholders.<br>• Provide self-learning modules and resources including:<br>○ A - Institutional analgesia and sedation guidelines education workshops, in-services, FLACC and WAT scoring educational videos<br>○ B – safety screening tool and checklist for SABT and extubation readiness workshops and online education tools<br>○ C – Institutional analgesia and sedation guidelines education workshops, in-services, RASS scoring educational video<br>○ D – delirium checklist and guideline education tools including non-pharmacological delirium management strategies workshops and in-services, CAP-D scoring educational video<br>○ E – early mobilisation and rehabilitation program education and training including activity level classification, mobility activities, assessment of physical function by cCPAx online examples<br>○ F – consumer information hybrid booklet, education and training around tools used to ensure family inclusion	6 months

	o G – education training around nutritional checklists/nutritional assessment tool used, importance of correct weight obtained<br>o H – education/training around tools to help personalise the patient for the clinicians (e.g. "getting to know you") and strategies to individualise care appropriately<br>• Measurement of baseline rate of PICU related complications (activity levels, over-sedation, withdrawal, delirium, PICU acquired weakness, ventilated patient days, VAP, CLABSI, immobility, CAUTI, fall, mediation error, PICU readmission, accidental line removal.<br>Pre-implementation Assessment.	
**Execute (Phase 3)**	• Integrate PICU Liberation into practice.<br>• Unit wide roll-out, one to three bundle elements at every one time.<br>• Regular assessment of above elements, these might be revised depending on feedback and evaluations. "Provide care as required“ to improve each element of the bundle.<br>• Post-marketing and education.<br>Pilot-Implementation, ongoing adaptation.	24 months
**Evaluate (Phase 4)**	• Evaluate Impact of PICU Liberation on practice.<br>• Regular evaluation -refine implementation and target education in response to feedback and control chart data implementation process and efficacy outcomes measurement.<br>• Re-measure bundle fidelity over time to determine maintenance of new practices.<br>• Assess impact of PICU Liberation on PICU related complication incidence and patient outcomes.<br>Assessment of feasibility, implementation success and impact.	12 months (overlap with execution phase as evaluation begins as bundle elements are rolled out)
